# Neuro–Immuno–Psychological Aspects of Chronic Urticaria

**DOI:** 10.3390/jcm12093134

**Published:** 2023-04-26

**Authors:** Katarzyna Tomaszewska, Aleksandra Słodka, Bartłomiej Tarkowski, Anna Zalewska-Janowska

**Affiliations:** Psychodermatology Department, Chair of Pulmonology, Rheumatology and Clinical Immunology, Medical University of Lodz, 92-213 Lodz, Poland

**Keywords:** urticaria, psychological stress, quality of life, neuroimmunology, inflammation, mast cells

## Abstract

Urticaria is a condition characterized by the development of itchy wheals (hives), angioedema, or both. The pathophysiology of chronic spontaneous urticaria (CSU) is still poorly understood. It is suggested that there is no dominant and independent mechanism of CSU; however, there are different immunological and non-immunological abnormalities that act simultaneously or/and follow each other resulting in clinical symptoms. The latest hypothesis points out that mast cells (MCs) to be activated via autoantibodies in autoallergic or autoimmune mechanism mediators released from degranulated MCs are responsible for the vasoactive and neurospecific effect in CSU. According to many clinical observations, it is suggested that psychological stress can be both a triggering factor in the onset of CSU and a modulating one in the course of the disease and therapy effectiveness. Of importance, the mechanistic background of the psychological stress response in the skin has not yet been fully elucidated. However, of note, a variety of inflammatory mediators, neuropeptides, and neurotransmitters facilitate this phenomenon. This review presents recent findings on the neuro–immuno–psychological aspects of CSU, highlighting an emerging role of neuro–immune interactions. It also points out the usefulness of psychological tools employment for the baseline diagnosis of perceived stress level and the presence of its symptoms. Furthermore, it proposes the implementation of non-invasive interventions to reduce psychological stress and anxiety. A bio–psycho–social approach including psychological support and patient education seems to be as important as traditional pharmacotherapy for CSU. It facilitates the effective control of active disease and a prolonged remission time in this disease.

## 1. Introduction

### 1.1. Characteristics and Epidemiology of Chronic Urticaria

Chronic urticaria (CU) is a condition characterized by the development of itchy wheals (hives), angioedema, or both, with reoccurring symptoms for more than six weeks [1]. The lifetime prevalence of all types of urticaria is usually below 10% according to different reports, whereas CU develops only in approximately one-fourth of these individuals. Of the total CU patients, one-third suffer from both hives and angioedema, 30–40% present isolated hives, and approximately 10% show isolated angioedema. The natural history of the disease has a very wide range. Approximately half of the patients follow a three-month self-limited evolution, and within a year the disease resolves in almost 80% of them. However, in more than 10% of the patients, a disease duration of 5 years or longer is expected. Females are affected at least twice as often as males, and most patients are over 20 years of age. In children, the prevalence varies from less than 1% to almost 5%, largely depending on the methodology employed by the researchers [2].

The purpose of this review is to summarize the findings on the neuro–immuno–psychological aspects of urticaria, regarding the bio–psycho–social model of patient care. The pathophysiology of urticaria is still poorly understood, although better knowledge of abnormalities in neuroimmune cutaneous response is the key to understanding the stress-related mechanism in this condition.

### 1.2. Current Insight into the CSU Mechanism

The pathomechanism of chronic spontaneous urticaria (CSU) is not well established. It is suggested that there is no dominant and independent mechanism of CSU; however, there are different immunological and non-immunological abnormalities that act mutually or/and follow each other resulting in clinical symptoms of CSU [3]. Undoubtedly, for all types of urticaria, the major players of the disease and wheal formulation are mast cells (MCs) and mediators released from these cells. Still, antihistamines constitute the first-line therapy in CSU; however, they are not equally effective in all cases [4]. Light and electron microscopy revealed degranulated MCs in the dermis following wheal appearance. According to some reports, increased numbers of skin MCs are detected in lesional and non-lesional skin in CSU and other types of urticaria; however, the results concerning the disposition of dermal MCs in urticarial patients are somewhat conflicting [3,5]. The activation of dermal MCs leads to immediate cell degranulation and the release of preformed mediators stored in granules, such as histamine, heparin, serotonin, chymase, tryptase tumor necrosis factor-α (TNF-α), nerve growth factor (NGF), and many others. Activated MCs also secrete de novo synthetized arachidonic acid metabolites including leukotrienes (LTs), prostaglandins (PGs), and a platelet activation factor (PAF) into the tissue. A wide range of cytokines and chemotactic agents including interleukins (ILs) such as IL-1, IL-4, IL-5, IL-6, IL-8, IL-10, IL-31, IL-33, macrophage inflammatory protein-1 (MIP-1), granulocyte–macrophage colony-stimulating factor (GM-CSF), transforming growth factor β (TGF-β), vascular endothelial growth factor (VEGF), fibroblast growth factor (FGF), TNF-α, and C–C chemokine ligand 2 and 5 (CCL2 and CCL5) are also released upon the MCs’ activation [5,6,7,8]. The vasoactive properties of the MCs’ mediators induce increased vasodilatation, an up-regulation of adhesion molecule expression, higher vascular permeability, and plasma extravasation. As a consequence, inflammatory cell/protein accumulation is observed in the affected skin. Importantly, some of the MCs’ mediators including substance P (SP), NGF, and vasoactive intestinal peptide (VIP) can interact with peripheral nerve endings and activate sensory nerves. Cross-talk between MCs and neuronal tissue is the fundamental point of the stress-induced skin neurogenic inflammation further discussed below. The abundance of neutrophils, eosinophils, basophils, CD4+ lymphocytes, as well as monocytes is detected in urticarial wheal. Furthermore, alternations in serum circulating T cell subtypes and their activities were observed in CSU vs. healthy subjects (imbalance in the proinflammatory Th17 cells and Treg cells). Skin biopsies revealed a higher expression of IL-4, IL-5, IL-25, IL-33, and thymic stromal lymphopoietin (TSLP) in urticarial wheal and non-lesional skin. These cytokines can promote a Th2-related response as well as amplify chronic inflammation and angiogenesis. On the other hand, an elevated expression of interferon γ (IFN-γ) in affected skin was detected, indicating a mixed Th1- and Th2-skewed polarization of skin immune response in CSU [3,4,5,9,10,11].

It is not entirely understood (i) what is the major stimulator of MC activation in the onset of CSU and (ii) which MC receptors are mainly involved in CSU-related activation. The proposed hypothesis points at MCs’ activation via autoantibodies, i.e., IgE specific to self-antigen (autoallergic mechanism) or IgG1/IgG3/IgM specific to IgE or its high-affinity receptor FcεRI (autoimmune mechanism). The autoimmune nature of the disease is supported by a deficit in T regulatory cell (Treg) activity in CSU. Tregs are generally responsible for suppressing the autoreactive immune response. It is worth remembering that FcεRI-mediated stimulation is not the only possible mechanism of MC activation considering the varied repertoire of MCs’ receptors. According to the stress-induced reaction in the skin, MC receptors specific to neuropeptides, neurotransmitters, and hormones are of special importance. There is a growing body of research on the potential role of other factors in CSU pathological cascade reaction, i.e., mediators other than histamine recruited inflammatory cells (e.g., basophils, neutrophils, and eosinophils) and skin-related cells, especially keratinocytes as a source of proinflammatory cytokines and activation of the coagulation system. The genetic background of CSU was also confirmed (alternations in HLA-DR4 and HLA-DQ8 gene expression) [3,4,12]. Interestingly, the urticarial lesion was reported to be the second most common skin-related symptom of COVID-19, which could also indicate the potential role of infectious agents in the course of CSU [13].

### 1.3. Urticaria affects the Quality of Life and Psychological Functioning

CSU is a stress-modulated condition in which the outcome of conventional treatment is often suboptimal. A bio–psycho–social approach including psychological support and patient education seems to be as important as traditional pharmacotherapy. It facilitates effective control and a prolonged remission time of the disease. During the COVID-19 pandemic and massive isolation, excessive fear, stress, and anxiety among people were reported. Patients suffering from chronic conditions, such as CSU, additionally experienced insufficient control of the disease. Beyaz et al. [14] have documented that urticarial activity score 7 (UAS7) during the pandemic time was higher compared to the pre-pandemic period. Furthermore, authors have observed that UAS7 was positively correlated with the Fear of COVID–19 Scale (FCV-19), depression, anxiety, and stress subscale score.

The association with psychological stress indicates the potential role of the neuroendocrine system in the etiopathogenesis of urticaria. However, it is still poorly understood how psychological stress interferes with the skin immune system in CSU. Although the role of psychological factors in development and aggravation is not fully confirmed, the psychosocial impact of CSU is widely accepted [15,16,17,18,19,20,21,22]. The struggle of chronic disease, prolonged treatment, and multiple consultations with different practitioners (often resulting in fatigue, work absence, or lowered school performance) [16,17] leads to a deterioration in psychological functioning. Patients suffering from urticaria manifest significantly higher scores in somatization, obsessive–compulsive disorder, interpersonal sensitivity and depression, anxiety, and stress levels [18,19,20,21]. A positive correlation between the severity of the disease and poor psychological wellness or lower quality of life is also frequently reported [22].

The wellbeing and quality of life of CSU patients is also affected by their quality of sleep. Subjective symptoms such as itching and pain can have a significant impact on sleep quality in patients with chronic skin diseases [23]. At the same time, sleep alterations (i.e., shortening of sleep; frequent awakenings during the night) lead to further deterioration in quality of life together with fatigue and frustration. In addition, patients with CSU are more likely to suffer from sleep disorders such as obstructive sleep apnea syndrome or sleep-disordered breathing [24,25]. As a consequence, there is an even greater psychological burden on the patient and, as a consequence, a higher risk of an exacerbation of CSU symptoms.

## 2. Psychological and Biological Aspects of Stress Reaction

### 2.1. Neurobiology of Stress Reaction

The regulation of the body adaptive response to stress involves mainly the hypothalamic–pituitary–adrenal (HPA) axis, and the sympathetic–adrenomedullary (SAM) system. The HPA axis plays a key role in maintaining body homeostasis and the body response to stress. Stress results in a corticoliberin-releasing hormone (CRH) release from the hypothalamus. This information is then transmitted to the anterior lobe of the pituitary gland, where the secretion of the adrenocorticotropic hormone (ACTH) takes place. This phenomenon leads to the stimulation of cortisol release into the blood from the adrenal cortex. An increased cortisol level leads to the inhibition of CRH and ACTH secretion by a negative feedback loop [26,27]. The importance of the HPA axis is mainly based on the action of cortisol. In stressful situations, cortisol is secreted as a body defense response. Cortisol reduces the inflammatory response, stimulates gluconeogenesis, and is responsible for protecting the body from an excessive immune response [26]. The SAM system represents another major immunoregulatory system. In general, SAM activation mediates short-term effects with rapid responses, whereas the HPA axis activation leads to short- and long-term effects [28,29]. Interactions of these two major stress systems (SAM and HPA) occur at several levels, functioning cooperatively and/or sequentially, acting in opposite ways in most visceral organ targets.

Furthermore, the response to physical and/or psychological stressors involves a rapid physiological adaptation mediated mainly by catecholamines. An epinephrine (E) and norepinephrine (NE) release from the adrenal medulla causes general physiological changes in order to prepare the body for a “fight-or-flight” reaction. E and NE effects include maintaining alertness, metabolic actions (increased glucose via glycogenolysis and gluconeogenesis, lipolysis, increased oxygen consumption, and thermogenesis), and cardiovascular actions [28].

The growing body of evidence shows the significant role of brain and gut interactions in stress response. The brain–gut axis consists in bidirectional communication between the central and the enteric nervous system and the intestinal one. The brain–gut axis creates a link between the emotional and cognitive centers of the brain with peripheral gut functions [30]. Mast cells are important effectors of the brain–gut axis. MCs translate the stress signals that have been transmitted through the brain–gut axis into the release of proinflammatory mediators. The latter can stimulate nerve endings and further affect afferent nerve terminals and change their perception, affect intestinal motility, increase intestinal hyperpermeability, and in susceptible individuals modulate the inflammation. Interestingly, MCs secrete a wide range of neurotransmitters and proinflammatory cytokines and express surface receptors for CRH demonstrating an important link between these cells and the stress response [31,32]. Furthermore, gut microbiota may participate in the modulation of the brain–gut axis and thus in the stress response. It was shown that microbiota composition may change as a result of the stress response leading to gut dysbiosis. The latter is especially noticed when permanent or repetitive exposure to stress situations is encountered. On the other hand, the individual stress response and the ability to adapt to stressful factors may depend on gut microbiota composition. That is why the treatment and care of patient suffering from stress-related diseases such as CSU should also focus on the restoration of gut microbiota composition. Applying a diet “friendly” to the gut microbiome or using specific oral probiotic strains, mainly the Lactobacillus family also called psychobiotics should be considered. Of note, more and more data, including clinical trials, indicate the benefits of psychobiotics supplementation (probiotics and prebiotics) on human cognitive function and the reduction of cortisol response. Research concerning the gut–microbiota–brain axis is a promising direction with great opportunities, although still much remains to be discovered in this field [33,34,35].

### 2.2. Stress as a Transaction; Acute and Chronic Stress

Psychological stress is strongly linked to the individual experience of difficult situations in everyday life encountered by all representatives of *Homo sapiens*. The situation of illness, i.e., the situation of being sick, including CSU, can be treated by the individual as a stressor and thus leads to psychological strain. The important point is that the psychological stress response to CSU will not be significantly different from stress experienced in other situations. Therefore, the mechanism described further on is universal and additionally reflects the process of stress in CSU patients. However, what is important for the emergence of a stress response is not the mere occurrence of the situation itself, but the course of the so-called stress transaction, which is the result of the *Homo sapien’s* relationship with the environment. The stress transaction takes place in two phases: the primary and secondary appraisals. The primary one involves an evaluation of the situation and identification of potentially stressful (threatening or difficult) events. The secondary appraisal involves an analysis of the demands and resources available to the individual in this particular situation. If in the course of these assessments, which are often unconscious and automatic in nature, the situation is judged to be potentially threatening and the individual resources are deemed insufficient to meet the demands of the situation, a stress response will occur, and the level of psychological stress will increase. It should be noted that this process is subjective in nature, and the same situation can be assessed in different ways depending on the course of the stress transaction. Throughout the process, it is also important for the individual to identify the possibility of taking action to remove the cause of the stress or its consequences: the implementation of a coping process [36].

According to Lazarus and Folkman’s theory, coping is a constantly changing cognitive and behavioral effort to manage specific external and internal demands evaluated by an individual as stressful, i.e., burdening or exceeding his or her own resources. Each individual has its repertoire of preferred coping styles, among which problem-focused (e.g., planning; active coping), emotion-focused (e.g., positive interpretation), or avoidance-focused (e.g., denial) styles can be distinguished. Problem-focused and emotion-focused strategies are complementary, and their employment leads to effective behavior in a stressful situation. If an individual has a positive attitude in a stressful situation and is able to regulate the level of arousal (emotion-focused coping) it helps to motivate and makes it easier to take action to eliminate the problem (problem-focused coping) [37].

Of importance, stress is not an explicitly negative and harmful phenomenon. It can be differentiated into positive stress (eustress) and negative stress (distress). The prerequisite for eustress is that the stressful situation is treated as a challenge and that the demands of the environment are assessed in favorable–positive terms, which inspires enthusiasm, hope, and motivation for positive change. Distress is the result of evaluating the stressful situation as threatening. It leads to impaired functioning, described most often as angry, tiring, excessive, prolonged, and thus harmful. It is a type of reaction that makes it difficult to perform tasks and control emotions, negatively affecting health and well-being [38]. It is also important to distinguish between acute stress, which is short-lived, occurs as a result of single situations or random events, and is usually adaptive, and chronic stress, which results from the experience of persistent difficult life situations or multiple episodes of acute stress. The experience of chronic stress can lead to permanent changes in the nervous system and results in a significant deterioration of psychological functioning, leading to psychological depression, anxiety, and somatic disorders [39].

### 2.3. Methods of Stress Level Diagnosis

Due to the highly subjective nature of the stress transaction, it is impossible to determine a universal level of stress occurring as a result of the disease for all the patients. Physiological, biochemical, and psychological methods can be used to measure the level of stress. All of the above methods have their advantages and disadvantages.

Physiological stress measurement mainly focuses on monitoring parameters such as heart rate, blood pressure, respiration rate, and changes in skin electrical resistance. The use of these measures will be particularly useful in acute stress situations but is not recommended in clinical practice and the evaluation of the actual stress level experienced by chronically ill patients [40].

Biochemical assessment involves mainly the evaluation of adrenal cortical hormones in the blood, urine, or saliva. An increase in catecholamines indicates the effects of short-term stress. An elevated concentration of corticoids (e.g., cortisol) and a low level of dehydroepiandrosterone sulfate (DHEA-S) accompanies chronic stress. However, the literature data on serum cortisol levels in CSU are sparse and conflicting [40].

Psychological methods to evaluate stress levels are of a questionnaire’s nature. They examine various aspects directly or indirectly related to stress. Popular questionnaires that directly indicate patient stress levels are the following: *Perceived Stress Scale* (PSS10), which measures subjective feelings of stress and the *Depression, Anxiety Stress Scale* (DASS) which is for measuring the intensity of perceived symptoms of depression, anxiety, and stress [41]. Of importance, due to the subjective nature of the stress transaction, we do not recommend the use of scales based on indicating experienced stressful situations such as the *Social Readjustment Rating Scale* (SRRS) when working with chronically ill patients.

Apart from questionnaires based on the direct measuring of stress levels, additional methods can be introduced to further enhance the evaluation of psychological functioning. The *Coping Orientation to Problems Experienced* (COPE) [37] and the *Coping Inventory for Stressful Situations* (CISS) [42] allow an assessment of the individual preference toward the use of different coping styles. The above questionnaires are used to assess tendencies to take actions that lead to effective coping with stress. Furthermore, the *Hospital Anxiety and Depression Scale* (HADS) [43] and the *State and Trait Anxiety Inventory* (STAI) [44] measure the level of emotions and emotional states typically associated with chronic stress, i.e., depression and anxiety. The aforementioned tools are used both for research purposes and in clinical practice. It is worth mentioning that due to various regulations worldwide, access to some of the mentioned questionnaires may be restricted to psychologists and psychiatrists only.

## 3. Stress-Induced Skin Reaction in the Course of CSU

### 3.1. Stress and Skin Diseases

**Figure 1:**
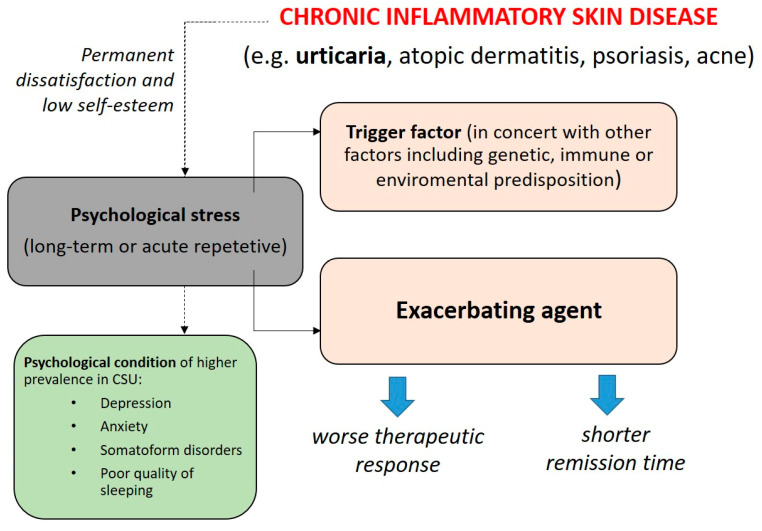
The hypothesis of bidirectional interactions between psychological stress in chronic skin diseases including chronic spontaneous urticaria (CSU) [12,50].

Many clinical observations confirm that there is a link between the onset and exacerbation of inflammatory dermatoses and a high exposure to psychological stress [45]. Regardless of the urticaria type, increased psychological stress is closely related to worsening and more frequent relapses of the disease [46]. Bidirectional interactions between psychological stress and chronic inflammatory skin diseases are presented in Figure 1. Of note, patients suffering from CSU are especially vulnerable to stress-induced alternations because the triggering agent of the disease remains unclear; therefore, signs and symptoms appear unexpectedly and spontaneously during life activities exerting continuous tension on the patient. Such a situation creates a vicious circle for the patients. On the one hand, illness itself leads to a worsening of the psychological functioning of the patient, and on the other hand, illness itself is exacerbated by psychological factors related to emotions and stress. It is also worth noting that most studies show a correlation between disease severity and psychological factors, without indicating what is the effect and what is the cause [14,47,48]. This means that stress reduction activities can lead to CSU symptoms’ alleviation. Therapeutical support and caring executed by an interdisciplinary medical team could lead to improvements in both patients’ skin disease severity and psychological functioning thus breaking the aforementioned vicious circle [49].

Long-term or frequent exposure to psychological stress affects every human organ, including the skin. The mechanistic background of the psychological stress response in the skin has not yet been well documented. Importantly, skin “battered” by dermatological inflammatory problems (e.g., urticaria, atopic dermatitis, psoriasis, and acne) is especially susceptible to stress-mediated influence. The reactive threshold of the skin response is lower than in healthy skin, probably due to a constant accumulation and arousal of immune cells, proinflammatory cytokines and chemokines in the lesional skin and non-lesional skin [3,51]. This phenomenon was also confirmed in the course of CSU. Furthermore, serum markers of systemic inflammation including C-reactive protein (CRP), IL-1β, IL-6, IL-18, TNF-α, IFN-γ, VEGF, and matrix metalloproteinase-9 (MMP-9) are in a higher concentration as compared to healthy controls. In CSU, this phenomenon can cause stress-induced exacerbations in the course of the disease [4,50,52,53,54]. Importantly, the cell receptor expression is not always constitutive. That is why a particular receptor may appear in response to an inflammable-specific milieu. For example, an elevated expression of corticotropin-releasing hormone receptor 1 (CRH-1R) is observed in CSU patients as compared to healthy individuals, indicating that CSU may be exacerbated by psychological stress via HPA axis stimulation [55]. CSU’s relation to stress was also linked to the activation of the opioid system in the chronic stage of the disease. Of importance, an elevated level of β-endorphins in CU patients vs. healthy subjects was also documented [56].

### 3.2. HPA Axis and CSU

The early stage of an acute stress reaction results in the temporary stimulation of the central HPA axis, which leads to stress hormone secretion, including CRH, ACTH, and cortisol. Cortisol is a natural glucocorticoid that acts as a specific inhibitor of the excessive immune response. After adaptation to an acute stressor, the HPA axis returns to normal activity due to a negative feedback mechanism mediated mainly by cortisol. It is suggested that some cytokines such as IL-6 and IL-18 may also play an important role in HPA axis modulation and control [57]. It is noteworthy that chronic stress or repetitive episodes of acute stress results in the dysregulation of the HPA axis which in turn leads to a lack of negative feedback control (fatigue/exhaustion of HPA). This may explain the increased levels of CRH, IL-1β, IL-6, IL-18, and other proinflammatory agents during the long-term exposition to stress that is observed in CSU [11,50,52]. Bearing in mind the systemic increase of some proinflammatory factors in CSU, it should be noted that brain homeostasis may be also disrupted in CSU patients causing an altered reaction to stress. However, more research data are needed to gain better knowledge on the mechanistic background of this phenomenon. It was observed that peripheral inflammation and MCs’ activation can alter the blood–brain barrier permeability (BBB). BBB is formed by endothelial cells, a basement membrane, astrocytes, and pericytes that separate the central nervous system (CNS) from peripheral circulation. Under normal physiological conditions, the BBB restricts the entry of peripheral immune cells into the CNS through a low expression of leukocyte adhesion molecules. However, in pathological conditions such as an inflammable-specific milieu, the above situation is interrupted. As a consequence, BBB becomes hyperpermeable through loss/alterations of its integrity. It should be underlined that this phenomenon also refers to the gut permeability-causing imbalance in microbiome and dysfunction of the local immune system. Inflammatory factors secreted by immune cells, as well as reactive oxygen species (ROS) and MMPs promote immune cells’ migration into the CNS and increased BBB permeability development. Thus, another vicious cycle could be formed [58].

Interestingly, the results of serum cortisol concentration in CSU patients compared to healthy subjects are inconsistent. Many studies have found no difference between CSU patients and controls according to basal cortisol levels [57,59]. On the other hand, Varghese et al. [52] have indicated lower cortisol levels in patients with CSU compared to controls. This result was negatively correlated with urticaria severity measured by UAS7, stress level, and disease duration. Vurgun et al. [57] have not found any difference in terms of cortisol concentration between the control group and CSU patients. However, the authors revealed a lower level of DHEA-S and higher cortisol/DHEA-S ratio in CSU patients. They further concluded that examining the ratio of cortisol/DHEA-S rather than hormones levels alone may be a useful tool for the evaluation of the HPA axis functions in an individual.

The skin appears to have the equivalent of an HPA axis with a similar cascade of hormonal response. Receptors for CRH (CRH-R1 and CRH-R2), urocortin, cortisol (glucocorticoid receptor-GR), ACTH (melanocortin receptors MC1R and MC2R), α-melanocyte-stimulating hormone (α-MSH), and β-endorphin are expressed on normal skin cells. Furthermore, an elevated expression of CRH-1R was observed in urticarial lesions. CRH-R1 is a major stress-related receptor constitutively expressed on the cells located in the epidermis, dermis, and subcutis, including MCs [54]. Acute stress and the intradermal administration of CRH stimulate skin MCs and increase vascular permeability in a CRH-R-dependent manner [55]. The association of CRH with mast cell degranulation has been also proven in rats [60]. CRH may also induce IL-6 and IL-18 release from keratinocytes and MCs [61]. Moreover, both CRH and ACTH were shown to activate basophils in humans [62]. Importantly, a skin cell can secrete stress-related hormones by itself and thus participate in local skin inflammation [54]. It should be emphasized that the skin HPA axis has its own, unique regulatory mechanism driven by cross-talk between nerve endings, resident skin cells/structures, and immune cells recruited from surrounding vessels (neuro–immune–cutaneous circuit). That is why a stress-induced skin reaction is difficult to interpret, especially if it concerns the reaction of the affected skin such as in CSU.

Konstantinou et al. [11] in their meta-analysis documented that current studies concerning stress-related responses in the course of CSU are focused mainly on (i) the assessment of the serum concentration of specific mediators including neuropeptides and pro-inflammatory agents, such as IL-18, CRP, neuropeptide Y (NPY), SP, stem cell factor (SCF), VIP, calcitonin gene-related peptide (CGRP), and NGF and (ii) the observation of the skin wheal and flare reaction after an injection of stress-related hormones and mediators, additionally before and after a specific CSU treatment.

Many researchers have observed some differences in the serum levels of neuropeptides and hormones in CSU patients compared to healthy controls. The results concerning SP serum concentration seem to be the most consistent. Basak et al. [63] documented that SP and stem cell factor (SCF) but not CGRP, VIP, NPY, and NGF were elevated in CSU patients as compared to healthy subjects. Metz et al. [64] have also reported that serum SP concentration was higher in CSU patients, and furthermore, the SP level correlated with the severity of CSU. Research performed by Zheng et al. [65] revealed that SP serum concentration was higher in CSU patients, and these subjects have an elevated number of basophils expressing NK-1R (receptor for SP) compared to controls. Furthermore, basophil stimulation with SP was stronger in CSU patients. Other studies revealed that CU patients have stronger wheal and flare reactions mediated through an injection with CGRP and SP [66]. SP is a stress-related pro-inflammatory neuropeptide that is released from cutaneous peripheral nerve endings. SP is the key mediator in connecting the brain to the hair follicle by stimulating mast cell degranulation and increasing macrophage infiltration [67]. Increased levels and cutaneous response to SP may lead to wheal and flares formation [68]. SP can also induce the expression of functional CRH receptor-1 in human mast cells [69]. SP may coordinate the interaction between the HPA axis and the sympathetic nervous system and can be a possible link between stress response and urticaria. SP is a crucial mediator in skin neurogenic inflammation. Receptors specific for SP are expressed on MCs (NK-1R and MRGPRX2), and this expression is up-regulated in severe CU [51]. It is suggested that SP may act as a trigger factor in the course of CSU; however, more research is still needed.

### 3.3. Neuro–Immuno–Cutaneous Circuit and Its Potential Implication in CSU

There is a link between the immune and neuroendocrine system and the skin. Neuronal tissue including autonomic and sensory nerves is widely distributed over all skin layers remaining in direct contact with resident skin and newly recruited skin cells such as leukocytes. Skin cells respond to nerve-induced stimulation via specific neuropeptides receptors. Elements of the neuro–immune–cutaneous circuit share many common mediators, i.e., hormones, cytokines, neuropeptides, and neurotransmitters. Skin cells and nerves become a target for many stress-related mediators; however, they themselves secrete various neurohormonal factors as well. This mutual communication actively regulates tissue inflammation and the maintenance of homeostasis under physiological conditions. However, in some skin pathologies including urticaria, cutaneous neurogenic inflammation develops. Many studies documented that wheal and flare reaction mediated by the intradermal injection of neuropeptides is significantly larger and longer lasting in patients with CU. The concept of neurogenic inflammation includes chronic increased vascular permeability, leukocyte infiltration, and protein extravasation caused mainly by neuropeptides released due to local and systemic HPA axis stimulation and acting in concert with proinflammatory agents [7,70]. Skin neurogenic inflammation reflects clinical observations that psychological stress may influence the course of inflammatory skin disease.

**Figure 2:**
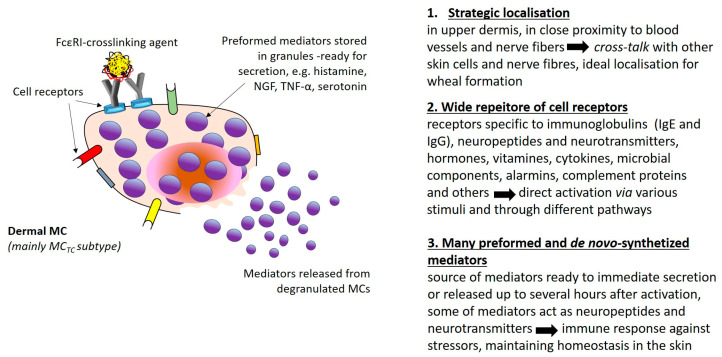
Mast cell as a link in the neuro–immune–cutaneous axis [72].

The role of MCs in the immunopathogenesis of CSU is unquestionable. Not without reason, dermal mast cells are regarded as a link in the neuro–immune–cutaneous axis also in CSU. The most crucial features of MCs’ biology are presented in Figure 2. It is worth remembering that every stimulation of MCs that leads to cell degranulation is inextricably linked not only to vasoactive and proinflammatory effects but additionally to sensory nerve stimulation.

According to stress-related skin response, the wide spectrum of MCs’ cell receptors with a high affinity to neuropeptides, neurotransmitters, neurokinins, and hormones are of particular interest. The main MCs’ neurohormonal specific receptors are presented in Table 1. Some of these receptors are additionally expressed on cutaneous nerves including those specific to histamine, NGF, SP, CGRP, and neurokinin-1. Interestingly, MCs’ expression of mas-related G protein-coupled X2 receptor (MRGPRX2/MrgX2) is elevated in CSU patients as compared to healthy individuals. Taking into account that the serum concentration of SP (main ligand of MrgX2) is also higher in CSU patients, controlling MrgX2-induced cell activation seems to be of high importance in the effective treatment of the different types of urticaria [5,70]. Currently used biological drugs affect mainly IgE and decrease free IgE levels. Subsequently, IgE receptors (FcεRI) on cells are down-regulated. A complete response rate of omalizumab ranges from 26% to 83% as demonstrated in several studies including XCUISITE, ASTERIA, and the recent ligelizumab trial. Thus, there is a need to search and aim at new etiopathological pathways such as those related to neurohormonal factors in order to implement new biological treatment options in CSU [71].

**Figure 3:**
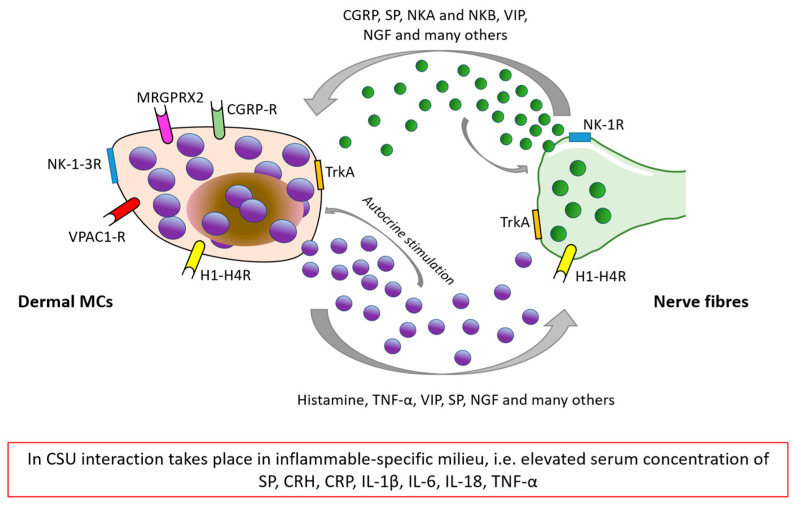
The possible cross-talk between MCs and nerve fibers as a basis of skin neurogenic inflammation in CSU [5,74]. **CGRP**: Calcitonin gene-related peptide, **NKA/NKB**: neurokinin A and B, **SP**: substance P, **CRH**: corticoliberin-releasing hormone, **CRP**: C-reactive peptide, **VIP**: vasoactive intestinal peptide, **NGF**: nerve growth factor, **TNF-α**: tumor necrosis factor-α, **NK-1-3R**: Neurokinin type 1–3 receptor, **TrkA**: Tropomyosin receptor kinase A, **H1R-H4R**: Histamine receptor type 1–4, **CGRP-R**: Calcitonin gene-related peptide receptor, **MRGPRX2/MrgX2**: Mas-related G protein coupled X2 receptor, and **VPAC-R**: (vasoactive intestinal peptide receptor).

Although nerve fibers remain in direct contact with many skin cells and structures (e.g., keratinocytes, sebocytes, fibroblasts, and hair follicles), their communication with MCs is well-documented and seems to be the main component of the stress-related neurogenic inflammation detected in affected skin [5,7,50]. Observations on the direct interaction between mast cells and local skin nerve fibers in human and animal models show that neural stimulation similar to stress-related leads to the secretion of neuropeptides capable of triggering MCs [15]. SP, CGRP, VIP, and neurokinins A and B (NKA and NKB) are of special importance regarding stress-related dermal MCs’ activation mediated by neurons. Moreover, many mediators secreted by MCs, such as histamine, ACTH, CRH, β-endorphins, neurotensin (NT), SP, NGF, VIP, and TNF-α may not only stimulate cutaneous nerves but also act in an autocrine manner on MCs’ activity and releasability [73]. Figure 3 demonstrates the possible cross-talk between dermal MCs and cutaneous nerve fibers in the skin affected by urticarial wheal.

## 4. Recommended Psychological Intervention in Stress-Induced Skin Diseases

Based on the above discussions and taking into account the role of psychological factors in CSU, it is worth considering the inclusion of stress reduction and psychological care as one element in the treatment of CSU [75,76]. In clinical practice, it is of importance to educate the patient and implement simple methods of stress and tension relief to reduce its harmful effects on psychological functioning. These are so-called anti-stress training or relaxation techniques, which—when used regularly—can help reduce the level of perceived stress by the patient. Examples of anti-stress training that can be used in clinical practice and with patients are shown in Table 2.

Authors should discuss the results and how they can be interpreted from the perspective of previous studies and of the working hypotheses. The findings and their implications should be discussed in the broadest context possible. Future research directions may also be highlighted.

The anti-stress training techniques listed in Table 2 are only some examples of the most popular techniques used in relaxation, tension reduction, and stress management support. It should be pointed out that not all anti-stress training is a substitute for professional therapeutic assistance, delivered by professional psychotherapists. Psychotherapy is effective as an adjunctive method for urticaria therapy and can be implemented when the patient is treated by an interdisciplinary team [79].

## 5. Conclusions

This manuscript highlights the complexity of stress and its association with MCs’ activity. Stress involves the HPA axis and SAM, but also the gut-brain axis, which provides bidirectional communication between the central and the enteric nervous system, linking the emotional and cognitive centers of the brain with peripheral intestinal functions. Mast cells are important effectors of those axes. Although FcεRI-dependent signaling is the main pathway in MC activation, FcεRI-independent mechanisms have also been suggested to play a role in CSU pathological cascade. Of special interest is the interaction between MCs and skin sensory nerve fibers as a source of many neuropeptides and neurotransmitters. Better knowledge of MCs’ stimulation via stress-related so-called neurohormonal receptors may be a key to the novel therapeutic approach to CSU. Recently, considerable progress has been made in developing new drugs that target MCs’ mediators or receptors. The possible inhibition of MRGPRX2 as a main molecule for substance P is being currently widely discussed. It is worth emphasizing that psychological stress in CSU not only acts as a pro-factor for the worsening of the symptoms of the disease but is also a consequence of the course of the disease. This leads to the emergence of a vicious circle, which is difficult to break. Therefore, the combination of psychological and traditional pharmacological therapeutic care as a permanent part of the treatment in CSU as well-done patient care executed within interdisciplinary teams to provide holistic care is highly expected.

## Figures and Tables

**Table 1:** Main receptors expressed on MCs involved in skin stress-related response [5,72,73].

Neurohormonal Receptors Expressed on MCs	Receptor Ligands
**NK-1R**-Neurokinin-1 receptor	SP-Substance P
**TrkA**-Tropomyosin receptor kinase A	NGF-Nerve growth factor
**MRGPRX2/MrgX2**-Mas-related G protein coupled X2 receptor	SP, VIP-Vasoactive intestinal peptide
**CGRP-1R, CGRP-2R**-Calcitonin gene-related peptide type 1 and 2 receptor	CGRP-Calcitonin gene-related peptide
**PAC1**- pituitary adenylate cyclase activating peptide type I receptor, **VPAC1-R, VPAC2-R**-vasoactive intestinal peptide receptor type 1 and 2	VIP, PACAP-Pituitary adenylate cyclase-activating polypeptide
**NK-2R, NK-3R**- Neurokinin-2 and 3 receptor, respectively	NKA-Neurokinin A (NK-2R), NKB-neurokinin B (NK-3R)
**Y1R, Y2R, Y3R, Y4R, Y5R**	NPY-neuropeptide Y
**H1R-H4R**-Histamine receptor type 1–4	Histamine
**CRHR-1 and CRHR-2**-Corticotropin-releasing hormone receptor	CRH-Corticotropin-releasing hormone
**MC1R and MC2R**-Melanocortin receptors	ACTH-Adrenocorticotropic hormone
**GR**-Glucocorticoid receptor	Cortisol
**MOR**-Mu opioid receptor	β-endorphin
**NTSR1**-Neurotensin receptor 1	NT-Neurotensin
**SSTRs**-Somatostatin receptors	SST-Somatostatin

**Table 2:** Commonly used–anti stress training/relaxation techniques [77,78].

Relaxation Technique	Description
Visualization	A method of imagining pictures, sensations, tastes, and smells without actual sensory experience. It can be conducted in receptive form, in which the practitioner himself allows the imagination to flow freely through his head, or guided, when the content of the visualization is given, and the patients’ task is to follow the instructions.
Jacobson training (Progressive muscle relaxation)	Helps to reach a state of relaxation by noticing the difference between “tensed” and “relaxed.” During its course, the patient alternately tenses and relaxes different muscles, starting with the head and ending with the legs and feet. This allows the person to consciously feel the relaxation of tensed muscles, even if the person exercising was previously unaware of the tension he or she was experiencing.
Mindfulness Training	Based on the conscious experience of stimuli coming from the environment or the patient body. It can be practiced in many ways; however, it is worth remembering to focus your attention on the “here and now” during the exercises, observe the stimuli that reach a person, and focus on one’s own feelings, but without trying to fight them or suppress those that are difficult at the moment. Mindfulness training helps one realize the relevance of the stimuli reaching the exercising person, has a calming effect, and aids relaxation.
